# Supplementary material for: New tools to study the interaction between integrins and latent TGFβ1
Source: bioRxiv. 2023 Jan 26:2023.01.26.525682. Preprint. [Version 1] doi: 10.1101/2023.01.26.525682 (PMC9901185; doi:10.1101/2023.01.26.525682)
Supplement: Supplement 1 [file NIHPP2023.01.26.525682v1-supplement-1.pdf]

bioRxiv preprint doi: https://doi.org/10.1101/2023.01.26.525682; this version posted January 26, 2023. The copyright holder for this preprint (which was not certified by peer review) is the author/funder, who has granted bioRxiv a license to display the preprint in perpetuity. It is made available under aCC-BY 4.0 International license.

Supplementary Material

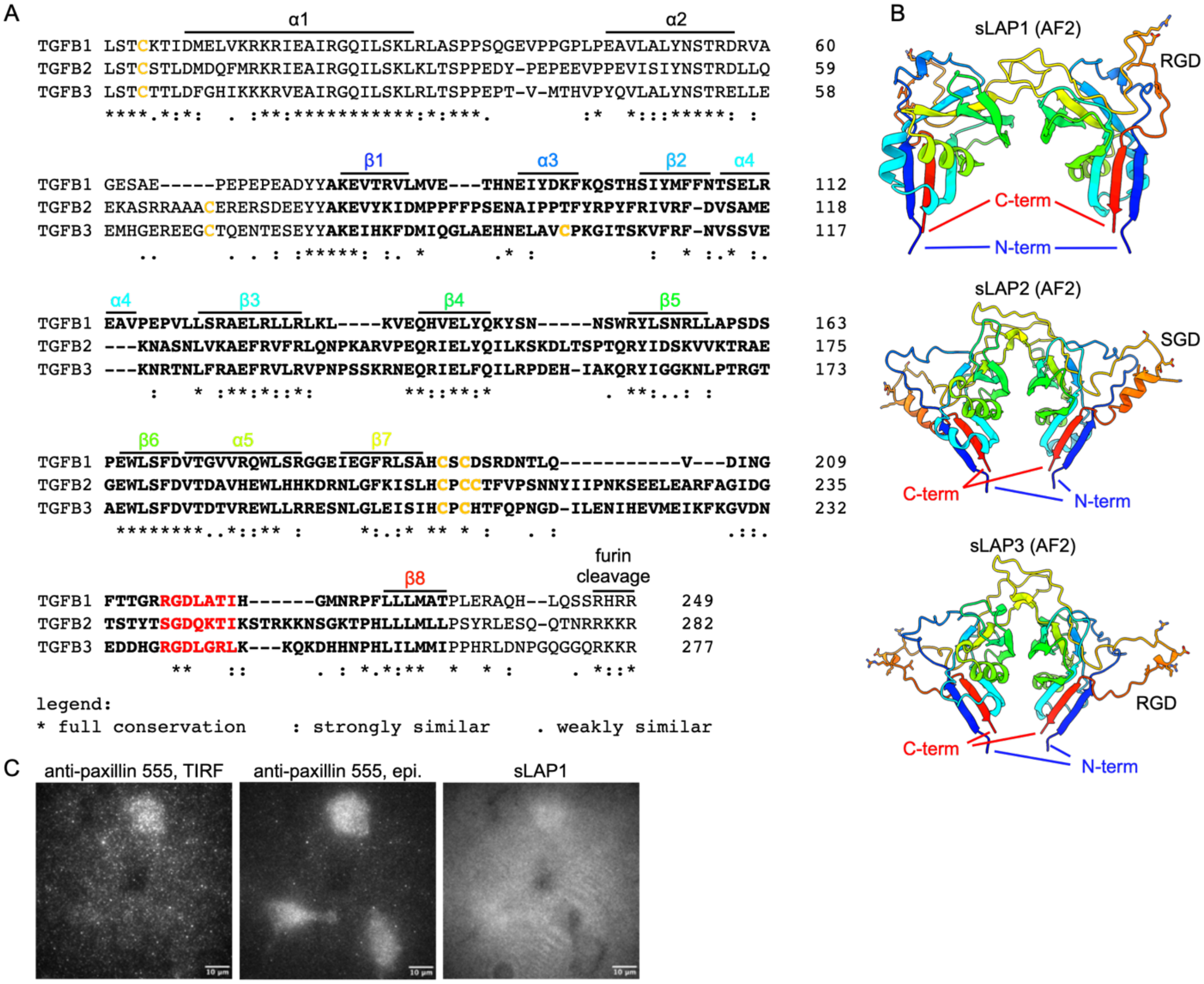

Supplementary Figure 01: (A) Sequence alignment of LAP parts of human TGFβ1-3. Cysteines are highlighted in yellow, regions used for sLAPs in bold, and RGD/SGD peptides in red. Numbering of secondary structures is based on 6GFF (Liénart *et al*., 2018); their color coding is according to sLAP1 shown in (B). (B) Alphafold2 predictions of sLAP1, sLAP2, and sLAP3. Secondary structures are rainbow colored with N-terminus in blue and C-terminus in red. Please note the dimerization of the LAP proteins via cysteines following β7 ("bowtie region"), the presence of the RGD/SGD peptides in a flexible loop, and the availability of both N- and C-terminus for adding molecular tags. (C) Mock-transfected NIH3T3 cells were cultured on sLAP1 coated substrates and were stained against endogenous paxillin. Please note the presence of cells in the field of view as indicated by the epifluorescent image of paxillin (middle panel), while these cells neither rearrange sLAP1 nor establish prominent focal adhesions.

bioRxiv preprint doi: https://doi.org/10.1101/2023.01.26.525682; this version posted January 26, 2023. The copyright holder for this preprint (which was not certified by peer review) is the author/funder, who has granted bioRxiv a license to display the preprint in perpetuity. It is made available under aCC-BY 4.0 International license.

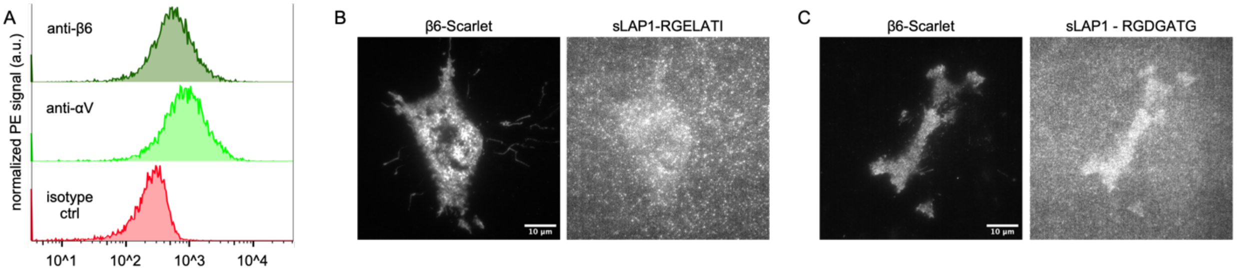

Supplementary Figure 02: (A) Flow cytometric analysis of surface expression of αV integrins and of β6 integrin. (B, C) β6-Scarlet expressing NIH3T3 cells cultured on sLAP1 substrates where the high-affinity RGD-LATI peptide was mutated to (B) RGE-LATI or (C) RGD-GATG.

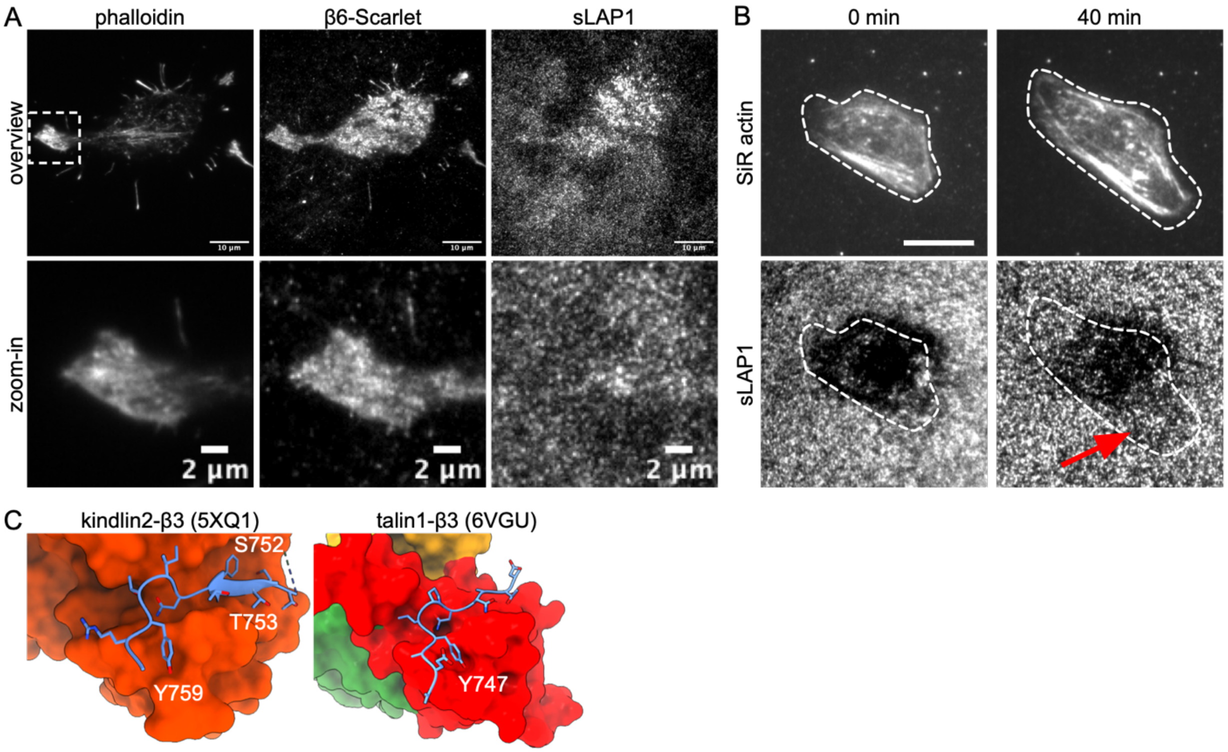

Supplementary Figure 03: (A) β6-Scarlet expressing NIH3T3 cells were cultured on sLAP1 coated substrate, fixed, and stained for actin with phalloidin 647. White box is shown as zoom-in in the lower row. (B) ASZ001 cells were incubated with 1 μM of SiR actin to visualize the actin cytoskeleton in living cells. Cells were imaged for 45 min every 2.5 min. Outline of cell at respective time points is shown as white dashed line. Please note the reduced sLAP1 fluorescence in areas occupied by the cell during the time lapse imaging (red arrow). Scale bar: 10 μm. (C) Structural analysis of integrin β3 tail (bright blue) binding to F3-domains of kindlin2 (PDB: 5XQ1, orange) or of talin1 (PDB: 6VGU, red; F2 in green, F1 in yellow). Please note the binding of the Tyr of the proximal and distal NxxY motifs to respective pockets in talin and kindlin. Also highlighted are S752 and T753 that are involved in forming a β-sheet with kindlin (see also Fig. 3B) and that have been shown to be important for integrin binding to kindlin (Soto-Ribeiro *et al.*, 2019).
